# Areas activated during naturalistic reading comprehension overlap topological visual, auditory, and somatotomotor maps

**DOI:** 10.1002/hbm.23208

**Published:** 2016-04-07

**Authors:** Mariam R. Sood, Martin I. Sereno

**Affiliations:** ^1^ Department of Psychological Sciences Birkbeck, University of London Malet Street London WC1E 7HX United Kingdom; ^2^ Experimental Psychology, Division of Psychology and Language Sciences 26 Bedford Way London WC1H 0AP United Kingdom

**Keywords:** cortical surface‐based analysis, fMRI phase‐encoded analysis, fMRI univariate analysis, reading comprehension, retinotopic mapping, tonotopic mapping, somatomotor mapping

## Abstract

Cortical mapping techniques using fMRI have been instrumental in identifying the boundaries of topological (neighbor‐preserving) maps in early sensory areas. The presence of topological maps beyond early sensory areas raises the possibility that they might play a significant role in other cognitive systems, and that topological mapping might help to delineate areas involved in higher cognitive processes. In this study, we combine surface‐based visual, auditory, and somatomotor mapping methods with a naturalistic reading comprehension task in the same group of subjects to provide a qualitative and quantitative assessment of the cortical overlap between sensory‐motor maps in all major sensory modalities, and reading processing regions. Our results suggest that cortical activation during naturalistic reading comprehension overlaps more extensively with topological sensory‐motor maps than has been heretofore appreciated. Reading activation in regions adjacent to occipital lobe and inferior parietal lobe almost completely overlaps visual maps, whereas a significant portion of frontal activation for reading in dorsolateral and ventral prefrontal cortex overlaps both visual and auditory maps. Even classical language regions in superior temporal cortex are partially overlapped by topological visual and auditory maps. By contrast, the main overlap with somatomotor maps is restricted to a small region on the anterior bank of the central sulcus near the border between the face and hand representations of M‐I. *Hum Brain Mapp 37:2784–2810, 2016*. © **2016 The Authors Human Brain Mapping Published by Wiley Periodicals, Inc**.

**Abbreviations**
ACv: Anterior cingulate visual area
ACa: Anterior cingulate auditory area
BUCNI: Birkbeck/University College London Centre for NeuroImaging
COPE: Contrast of parameter estimate
DLPFC: Dorsolateral prefrontal cortex
FEF: Frontal eye field
FEAT: FMRI Expert Analysis Tool
GFEAT: Group FEAT
HG: Heschl's gyrus
HRF: Hemodynamic response function
iCG: Isthmus of cingulate gyrus
PFC: Prefrontal cortex
POS: Parieto‐occipital sulcus
PP: Planum polare
PT: Planum temporale
SMA: Supplementary motor area

## INTRODUCTION

Topological (neighbor‐preserving) remapping is a key principle of organization of sensory and motor areas within the mammalian brain. In primary sensory and motor cortices, these representations initially reflect the spatial layout of the receptors; for instance retinotopic maps in visual cortex topologically encode retinal locations, tonotopic maps in auditory cortex represent positions along the cochlear hair cell line, which correspond to sound frequency, and somatotopic maps in the somatosensory cortex represent locations on the body surface. Disrupting these maps has been shown to affect subsequent sensory processing and behaviour [Kaas, 1997; Sperry, 1943]. Traditionally it has been assumed that topological mapping was limited to lower‐level (primary sensory and motor) cortex [Hebb, 1949]. But the entire cortex is characterized by the overwhelming predominance of local connections [Ercsey‐Ravasz et al., 2013; Lund et al., 1993; Schmahmann and Pandya, 2009]. The last few decades of electrophysiological investigations in monkeys and neuroimaging research on humans has shown that topological organization extends well into higher‐level (beyond primary sensory and motor) cortical areas [Felleman and Van Essen, 1991; Huang and Sereno, 2013; Sereno and Allman, 1991; Wandell et al., 2007]. In contrast to lower‐level maps, however, localized activity in higher‐level maps is affected as much by spatial attention as by the spatial characteristics of the stimuli [Saygin and Sereno, 2008]. In frontal cortex, there is evidence that topological maps may serve as a convenient method of allocating working memory, or maintaining pointers to specific content [Hagler and Sereno, 2006], even for tasks not overtly referencing position; for example, the exact areas that showed *more* robust activity during an *identity* two‐back task (in which location was ignored), than during a *location* two‐back task (in which identity was ignored) turned out to contain retinotopic maps. A significant role for topological maps in other complex mental operations has been suggested before [Simmons and Barsalou, 2003; Thivierge and Marcus, 2007], but direct neuroimaging evidence supporting this idea has been scant, and mostly confined to somatosensory cortex.

In the study presented here, we used fMRI to directly assess the extent of overlap between cortical regions involved in reading comprehension and those that have a topological sensory or motor map. It is generally assumed that the activation observed in temporal and frontal areas during reading falls beyond the bounds of sensory‐motor maps, but this assumption has not been explicitly tested across all modalities in the same group of subjects. Recent advances in cortical surface‐based mapping techniques have had less exposure in the language literature; for example, “sensorimotor” regions are often defined only by anatomical features or by basic, non‐attention demanding tasks. In this study, we look at the full extent of topologically mapped sensory‐motor regions that can be reliably detected using retinotopic, tonotopic and somatomotor mapping and assess where and by how much they intersect with brain regions involved in reading comprehension in the same subjects. To this end, each subject in our study participated in four separate fMRI sessions, where the sessions comprised a naturalistic reading comprehension task, retinotopic mapping, tonotopic mapping, and somatomotor mapping. A key goal of this study was to determine to what extent topological sensory‐motor maps, traditionally associated with low‐level sensory and motor processing are present in the cortical regions significant for a complex high‐level cognitive task such as reading comprehension. Although we have used the terminologies “lower‐level” and “higher‐level” in its traditional sense for ease of explanation, it is important to keep an open mind about “levels”: an apparently lower‐level area may be performing language‐specific functions while an apparently higher‐level area may be performing other functions besides language.

On the methodological front, we employed a fully surface‐based group analysis as opposed to volume‐based group analyses commonly used in language studies (merely displaying a 3‐D averaged result on an average surface gains none of the benefits of surface‐based averaging). The cerebral cortex has the topology of a 2‐D sheet. Many relevant dimensions (e.g., retinotopy, somatotopy, tonotopy) vary much more rapidly tangential to the cortical surface than they do perpendicular to the cortical surface, through the several millimeters of cortical thickness. Distances measured in 3‐D space between two points—but also used in standard pre‐fitting 3‐D smoothing—can substantially underestimate the true distance along the cortical sheet due to its folded nature [Fischl et al., 1999a, 1999b]. This artifactual within‐subject blurring is then made worse by 3‐D averaging of between‐subject variability in the secondary crinkling of cortical folding patterns. Surface‐based techniques make it possible to restrict smoothing to directions parallel to the cortical sheet, and to employ inter‐subject 2‐D alignment based on the patterns of sulci and gyri after secondary crinkles have been removed, which reduces both kinds of artifactual blurring and improves cross subject averaging [Fischl et al., 1999b]; this also provides a less biased estimate of overlap. Finally, siting language regions with respect to topological cortical maps provides a more precise way to compare activations across individuals and groups as well as studies. This is particularly important for refining functional localization in less well‐understood regions such as frontal cortex.

## MATERIALS AND METHODS

### Subjects

20 right‐handed native English speakers (nine women) participated in this study. The mean age was 28 (ranging from 19 to 58). All participants were neurologically healthy with normal or corrected to normal vision and normal hearing capacity. The experimental protocols were approved by local ethics committees and participants gave their informed written consent prior to the scanning session. The study required each participant to take part in four separate fMRI experimental sessions: Reading task, Retinotopic mapping, Auditory mapping and Somatomotor mapping. All 20 participants took part in Reading task and Retinotopic mapping experiments. 18 of the same participants took part in Auditory mapping and 17 of the same participants took part in Somatomotor mapping. The data from three participants in the Reading task and two participants in the Somatomotor mapping task did not meet the required quality criteria and were excluded from group results (discussed further in the Results section).

### Experimental Stimuli and Design

#### Reading experiment

The reading experiment consisted of a naturalistic reading task where comprehension blocks contained a short narrative passage in English. The experiment was a random‐order block design with three conditions and a central fixation screen. Each condition block was 16 sec long. During the experimental condition, a passage in English was presented one word at a time for 16 sec (64 words in total, average rate 4 words/sec). Each word was briefly presented on the screen at its natural reading position within the text. All other words appeared as grayed rectangles. The background screen was shown at 40% brightness (RGB: 102,102,102), the word was shown at 0% brightness (RGB: 0,0,0) and the rectangles were shown at 36% brightness (RGB: 92,92,92). The space between successive words/rectangles was set to 35% of the average word length. The exact display time of a particular word was made a piecewise linear function of the rendered word length. Vera.ttf, a san‐serif typeface was parsed by FreeType 2.4.11 and rendered and measured in OpenGL using FTGL 2.1.3. The minimum display duration of a word was clamped to 175 msec, which was 70% of the average word duration of 250 msec. Words with widths more than 70% of the average word width were then given linearly increasing display durations with a slope chosen so the total paragraph duration equaled the desired block length (16 sec). This resulted in virtually identical duration/word‐length slopes across different 64‐word paragraphs. The guided reading experience felt much more subjectively natural when duration was controlled by word length than when a fixed word duration was used. In order to control for low level visual processing, there were two other conditions.

**Figure 1:**
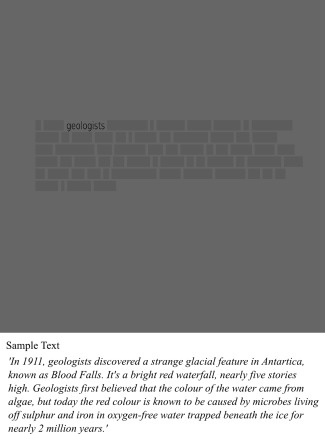
Reading stimulus‐English screen and sample English text.

The condition 2 consisted of the “Hindi” version of the English passage (simple substitution of characters from the font, lekhani_dynamic.ttf, not a translation, so rendered word lengths remained identical) using presentation mode and fixation durations identical to the English condition. In the third condition, Dot, a 0.5 deg visual angle dot instead of a word briefly replaced each of the grayed rectangles, again with the same varying fixation durations and rectangle lengths. The baseline condition (condition 0) presented a single central fixation dot. The experiment consisted of 4 runs, where each run was comprised of 32 blocks presented in a random order. Participants, who had no familiarity with “Hindi” were instructed to do their best to comprehend the English passages in all four runs and to follow the Hindi script “words,” or dot in the other two conditions. The passages were self‐contained and unrelated to each other. The level of comprehension achieved for the English passages was measured with a questionnaire afterward. The stimulus presentation technique used here, where each English word, Hindi word or dot was briefly presented in its natural reading position with all other words grayed out served several purposes. In the spirit of the classic attention study by Posner [1980], the subject's exogenous attention is automatically drawn toward each newly highlighted position, where the word or dot appears, in a manner very similar to natural reading. The subjects moved their eyes along with the highlighted word and reported a naturalistic reading experience, which was aided by a naturalistic (word‐length dependent) fixation duration. Additionally this presentation mode also ensured that participants made controlled eye saccades (and extremely similar eye movements across conditions) as opposed to uncontrolled eye movements if the passages were presented in its entirety. In order to further ensure that participants stayed attentive and made similar eye‐movements for all conditions, they were instructed to press a button when the color of an English word, a Hindi pseudo word, a dot, or the central fixation dot changed from black to off‐white (50% brightness, RGB 128,128,128) for an average of 0.25 seconds. The responses to button press events were logged and analyzed to assess the quality of task execution. Button press events were modeled as an extra regressor and used as an additional quality control check during data analysis. The English screen of the experiment and a sample English passage are shown in Figure 1. The stimulus was programmed in C/OpenGL/X11 (stimulus program available on request). An optimized random order of the conditions within each run was generated using AFNI's [Cox, 2012] RSFgen program. Prior to the scan, participants were shown a demo of the experiment (using different English passages). They were instructed to read with an intention to comprehend during the “English” condition, to move the eyes along with the “Hindi”/“Dot” word in Hindi and Dot conditions and to fixate at the central dot during the fixation condition. They were asked to perform the target detection task that occurred randomly across all four conditions. The participants were informed that the button press events would be logged to assess how well they followed the eye movement instructions (and performed the task) and that the comprehension of English passages would be assessed using a questionnaire after the scan.

#### Retinotopic mapping

**Figure 2:**
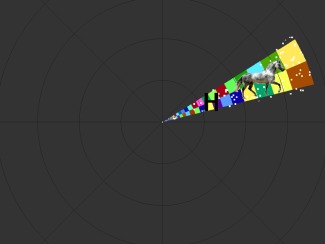
Retinotopic mapping stimulus.

In the retinotopic mapping experiment, we mapped polar angle using a phase‐encoded stimulus very similar to that used in previous recent work by Sereno et al. [2013]. The stimulus (Fig. 2) consisted of a continuously rotating thin wedge (18 deg wide) populated with a random‐colored checkerboard with 35% luminance contrast. The checkerboard was overlaid with white dot fields moving in 500 msec periods of coherent motion that extended slightly beyond the checkerboard wedge to 21.6 deg wide (each new flow period had randomly chosen contraction/dilation and clockwise/anti‐clockwise components, dots had 50% average luminance contrast), as well as two simultaneous asynchronous streams of random objects (tiffs of single objects with a transparent background, 0.5 sec duration, 0.1 sec gap) and random black letters (0.4 sec duration, 0.1 sec gap) placed at random eccentricities; both kinds of stimuli were scaled with eccentricity to fit within the confines of the 21.6 deg wide wedge, and their centers were rotated together with the wedge. The objects had an additional random radial (inward or outward, eccentricity‐scaled) motion. This polar angle mapping stimulus was designed to evoke activation in the maximum number of lower‐level and higher‐level visual areas. The participant was presented with the periodic stimulus (64 sec per full rotation cycle, eight cycles/run). 512 second runs (four in total) alternated between clockwise and anti‐clockwise rotation of wedges. Participants were required to fixate on the center dot at all times. Additionally they were instructed to monitor for occasional numbers (among the letters) and occasional upside down objects (among the right‐side‐up objects) to maintain a high and continuous level of peripheral attention to the entire wedge during central fixation.

#### Auditory mapping

In the auditory mapping experiment, we mapped tonotopically organized cortical areas using a frequency‐modulated stimulus taken unchanged from the previous study by Dick et al. [2012]. The stimulus consisted of bandpass‐filtered nonlinguistic vocalizations adapted from the Montreal Affective Voices [Belin et al., 2008], a series of recordings by actors (only male voices were utilized in the stimulus) producing sounds associated with a set of eight emotions. Each run consisted of eight 64 sec band‐pass‐filtered cycles, where the center filter frequency repeatedly logarithmically ascended from 150 to 9,600 Hz, or repeatedly descended from 9,600 to 150 Hz (no gap at the wrap‐around point). The session consisted of 4 runs alternating between ascending and descending frequency sweeps. During scanning, subjects were asked to monitor the stimuli and press a button whenever they heard laughter (laughter targets were distributed non‐periodically through the stimulus train). The band pass filter made this task challenging.

#### Somatomotor mapping

In order to reveal sensory and motor maps in primary and secondary somatosensory and motor cortical areas, participants were asked to move different body parts in response to periodic auditory cues (spoken name of the body part to be moved, rendered using Mac OS X text‐to‐speech with “Alex” voice) in as gentle and localized fashion as possible. Each run consisted of 8 cycles of 64 sec, and in each cycle participants successively moved 11 different body parts progressing from tongue to toe. Runs (four in total) alternated between movement cycles in each direction (from tongue to toe and toe to tongue). The stimuli were presented by an in‐house C/OpenGL program and the conceptual design is similar to that described in Zeharia et al. [2012]. The participants were allowed to familiarize themselves with the auditory cues and practice the controlled movements prior to the scan.

Auditory instructions were: “say tchuh‐tchuh”; “say pup‐pup”; “crinkle eyebrows”; “touch thumb index”; “wave wrist”; “contract biceps”; “pull in stomach”; “squeeze buttocks”; “contract quads”; “wave ankle”; “rub big toe”.

### Experimental Set‐up

For visual experiments (Reading task and Retinotopic mapping), the stimuli were projected into the bore using an Eiki LC‐XG300 XGA video projector onto a translucent direct‐view screen at the participant's upper chest level. All polar angles of the visual field were stimulated out to an eccentricity of at least 54 degrees of visual angle (much larger than the usual 8–12 degrees of all‐polar‐angles eccentricity achieved when a standard screen is viewed via a mirror). This avoids artifactual periodic modulation of voxels representing visual field locations beyond the outer edge of the stimulus due to surround inhibition. A black matte shroud situated just outside the bore blocked the beam from making low‐angle reflections off the top of the bore. The rear of the head coil was elevated with a wooden wedge and thinner bed cushions were used to help naturally tilt the head forward. For auditory and somato‐motor mapping, the stimuli were delivered binaurally using in‐house safety‐enhanced Sensimetrics (Malden) S14 earbuds and cushions. During all scanning sessions, memory foam cushions (NoMoCo Inc.) were packed around the head to provide additional passive scanner acoustical noise attenuation and to stabilize head position. Responses were made via an optical‐to‐USB response box (LUMItouch, Photon Control, Burnaby, Canada) situated under their right hand.

### Imaging Parameters

Functional images were acquired on a 1.5 T whole‐body TIM Avanto System (Siemens Healthcare), at the Birkbeck/University College London Centre for NeuroImaging (BUCNI), with RF body transmit and a 32‐channel receive head coil. For the first 16 fMRI sessions, images were acquired using the standard product EPI pulse sequence (24 slices, 3.2 × 3.2 × 3.8 mm, 64 × 64, flip = 90°, TE = 39 ms, TR = 2 sec), while the remaining 64 sessions used multiband EPI (40 slices, 3.2 × 3.2 × 3.2 mm, flip = 75°, TE = 54.8 ms, TR = 1 sec, accel = 4) [Moeller et al., 2011]. Individual scans had 260 volumes for standard EPI and 520 volumes for multiband EPI. To allow longitudinal relaxation to reach equilibrium, 4/8 initial volumes were discarded from each run for standard/multiband EPI. For each imaging session, a short (3 min) T1‐weighted 3D MPRAGE “alignment scan” (88 partitions, voxel resolution 1 × 1 × 2 mm, flip angle = 7^°^, TI = 1,000 ms, TE = 4 ms, TR = 8.2 ms, mSENSE acceleration = 2x, slab‐selective excitation) was acquired with the same orientation and slice block center as the functional data, for initial alignment with the high‐resolution scans used to reconstruct the subject's cortical surface. Two high resolution T1‐weighted MPRAGE scans (176 partitions, 1 × 1 × 1 mm, flip angle = 7^°^, TI = 1,000 ms, TE = 3.57 ms, TR = 8.4 ms) were acquired along with the fMRI sessions for cortical surface reconstruction (using FreeSurfer 5).

### Data Analysis

#### Anatomical image processing

For each subject, the cortical surface was reconstructed with FreeSurfer [version 5; Dale et al., 1999] from the aligned average of the two high‐resolution T1‐weighted MPRAGE scans. Both mapping data and reading data employ a cross‐subject surface‐based analysis stream that begins by sampling responses and statistics to individual reconstructed cortices (cross‐subject 3D averaging was not used at any point in the pipeline).

#### Analysis of phase‐encoded mapping data

The first level single subject data from each run was motion corrected and registered to the last functional scan (across all runs) using AFNI's 3dvolreg [Cox, 2012] program (using heptic interpolation). The functional images were registered with the high resolution MPRAGE using a multi‐stage registration pipeline. In the first stage, we generated a registration matrix to register the “alignment scan” (same block center and rotation as functional images, but only 3 min long) to the “high resolution” scan, since the contrast of these two scans was very similar. Each functional was registered to the “alignment scan” separately (these scans shared the same block center and rotation and this step was done to mainly correct any small variations between the four functional scans and the alignment scan). These registrations were done using FSL's FLIRT tool [version 5; Jenkinson and Smith, 2001; Jenkinson et al., 2002] and were then fine tuned using FreeSurfer's bbregister [Greve and Fischl, 2010]. The two registration matrices (functional ‐> alignment and alignment ‐> hi‐res MPRAGE) were combined to generate the 4x4 registration matrix that aligns functional EPI to high‐resolution MPRAGE (the matrix actually transforms 3D surface vertex coordinates to 3D EPI block index coordinates). The final registrations (EPI ‐> hi‐res MPRAGE) were visually checked to ensure accuracy. The time courses from all the runs were averaged (after time‐reversing the runs in the clockwise rotation for retinotopy, the upward frequency sweep for auditory, and the toe‐to‐tongue movement direction for the somatomotor map). The time reversed scans were time‐shifted to compensate for hemodynamic delay before averaging. The averaged time courses were analyzed using linear Fourier methods [Bandettini et al., 1992; Engel et al., 1994, 1997; Hagler and Sereno, 2006; Sereno et al., 1995], which can be exactly recast as a general linear model. Voxels preferentially responding to a particular point in the stimulus cycle will show higher amplitude at the stimulus frequency than at any other 'noise' frequency, after excluding (i.e., linearly regressing out) the three lowest temporal frequencies as motion artifact. For retinotopic data, the phase of this vector at the stimulus frequency indicates the polar angle of the stimulus location. For auditory data, the phase indicates a particular point on the stimulus frequency ramp. For somatomotor data, this corresponds to the location of the moved body part. The individual and group analyses utilized a complex‐valued cortical surface‐based stream that was previously described [Hagler et al., 2007; Huang et al., 2012; Sereno et al., 1995] and briefly summarized below.

A fast Fourier transform was first performed on the average time courses of each voxel. An *F*‐statistic value was obtained for each voxel by comparing the power at the stimulus frequency (eight cycles per scan) to the average power at the remaining frequencies after excluding the second and third harmonics of the stimulus frequency and one frequency above and below the first three harmonics. For individual subjects' activations illustrated below, the F‐statistic was thresholded at *P* < 0.001 (corresponding to *F*(2,232)=7.12 for subject 1 who was scanned on standard EPI with 256 time points and *F*(2,488)=7 for subject 2 and subject 3 who were scanned on multiband EPI with 512 time points). Surface‐based cluster size exclusion [Hagler et al., 2006] was used to correct for multiple comparisons with cortex surface clusters smaller than 30 mm^2^ excluded, achieving a corrected *P*‐value of 0.01.

Group analysis of phase‐encoded mapping data was performed using the methodology developed by Hagler et al. [2006] in which the real and imaginary components of the signal at the stimulus frequency were averaged across the subjects, preserving any phase information consistent across subjects (this is a vector average, which properly treats wrap around in the circular phase variable). This was performed by projecting each participant's complex‐valued phase‐encoded map to the FreeSurfer spherical atlas (using FreeSurfer mri_surf2surf), only performing ten steps of surface‐based smoothing (∼3mm FWHM in 2D) before vector averaging across subjects at each vertex in the common surface coordinate system. Second‐level surface‐based cluster size exclusion [Hagler et al., 2006] was used to correct for multiple comparisons, with vertex level F‐statistics thresholded at *P* < 0.01/*P* < 0.05 and cortical surface clusters smaller than 40 mm^2^/92 mm^2^ excluded, achieving a corrected p‐value of 0.05. We used the fsaverage “inflated_avg” surface for display (made by averaging inflated surface coordinates) instead of the fsaverage “inflated” surface (made by averaging folded surface coordinates and then inflating the average) because inflated_avg represents original average surface area better. This is because folding variations (sulcal crinkles) are removed before surface‐averaging, making inflated_avg, more appropriate for displaying surface‐averaged data (mesh defects in the north and south icosahedral poles of FreeSurfer5's inflated_avg were corrected before using it for display. Corrected and flattened inflated_avg surfaces are available here: http://www.cogsci.ucsd.edu/~sereno/.tmp/dist/csurf/fsaverage-adds.tgz).

For Somatomotor mapping data, in addition to the above analysis, we reanalyzed the data utilizing the subject‐ICA based noise reduction approach using ICA‐AROMA [Pruim et al., 2015] to reduce the motion artefacts and activations due to B0 deformations. Each subject's raw data was pre‐processed in FSL. Pre‐processing involved motion correction with MCFLIRT, intensity normalization and spatial smoothing using a Gaussian kernel of 5 mm full width at half maximum. We then used ICA‐AROMA to identify residual motion‐related artefacts. The identified motion components were removed from the original unsmoothed raw‐data (as smoothing the raw‐data disrupts the map structure) by means of a linear regression using the FSL program fsl_regfilt. The resultant data set was used as input for the phase‐encoded analysis pipeline described above. ICA‐AROMA was successful in reducing the noise related activations and group results utilizing this technique are depicted in the somatomotor mapping results described below. The original analysis (without noise correction using ICA‐AROMA) is also included in the Supporting Information Figures for comparison.

#### Analysis of Reading comprehension data

For the reading experiment, single subject fMRI data was motion corrected and skull stripped using FSL tools (MCFLIRT and BET). First level fMRI analysis was carried out by applying the General Linear Model (GLM) within FEAT using FILM prewhitening (FSL, version 5) with motion outliers (detected by fsl_motion_outliers) being added as confound regressors if there was more than 1 mm motion (as identified by MCFLIRT). A small number of scans with excessive motion above the threshold of 1 mm were excluded from analysis. High‐pass temporal filtering of the data and the model was set to 100 seconds based on the power spectra of the design matrices (estimated by cutoffcalc; part of FSL). Three main explanatory variables were modeled and controlled: Reading English text, viewing Hindi text and viewing dot “text” (stimulus duration of each block: 16 seconds). Button press responses to target color change events were modeled as the fourth condition of interest (stimulus duration modeled as 1 second). The stimulus waveforms were convolved with FSL's double gamma “canonical” hemodynamic response function (HRF) [Glover, 1999] to generate the main regressors. In order to capture slight deviations from the model, temporal derivatives of all explanatory variables convolved with double gamma “canonical” HRF were included. The registration from functional to anatomical (6 DOF) and standard space (12 DOF) was first done using FSL's FLIRT and further optimized using boundary based registration (bbregister; FreeSurfer) similar to the procedure for the phase‐encoded mapping data. A fixed effects analysis was performed across runs from an individual subject (usually four runs unless a run was excluded due to excessive motion/lack of attention as evidenced by poor performance on qualitative assessment/lack of response to the targets) to get group FEAT (GFEAT) results of first‐level contrast of parameter estimates (COPEs) and their variance estimates (VARCOPEs) in the standard space. Across‐subject group analysis was then carried out on the cortical surface using FreeSurfer tools. The GFEAT results of each subject were first sampled to individual cortical surfaces and then resampled to the spherical common average reconstructed surface (fsaverage). Surface‐based spatial smoothing of 3 mm FWHM was applied on the icosahedral sphere. A mixed effects GLM group analysis was performed on the average surface using the mri_glmfit program from FreeSurfer. Surface‐based cluster size exclusion utilized in phase‐encoded mapping data was used to correct for multiple comparisons with vertex level *T*‐statistics thresholded at *P* < 0.01, and cortical surface clusters smaller than 40 mm^2^ excluded, achieving a corrected *P*‐value of 0.05. Finally corrected significance values (*P* < 0.05) of reading activation were displayed on the average surface.

The single subject raw data was not spatially smoothed (both for phase‐encoded analysis and reading analysis). As with the phase‐encoded data, a 10 step (∼3mm FWHM) surface level smoothing was applied for final illustration of the results. Hence, 3D Gaussian random field based cluster correction provided by FSL was not appropriate for multiple comparison correction of the reading data. We have instead used the surface‐based cluster correction similar to that employed for phase‐encoded data analysis. The GFEAT results were sampled to their respective anatomical surface, thresholded at *P* < 0.001 (*Z* = 3.09) and corrected for multiple comparisons with cortex surface clusters smaller than 30 mm^2^ excluded, achieving a corrected *P*‐value of 0.01.

The target (font color change) presentation timings and the button press events were logged during the experiment and analyzed to assess the performance of the task. A target was considered as detected if there was a response (a key press event) within 1 second after the color change event had ended. For each participant, the number of targets detected and the mean response time in each condition were calculated. The cross‐subject mean response time and target detection rates were assessed for significant differences across conditions.

#### Overlap analysis

All overlaps were calculated using “original vertex‐wise area” in FreeSurfer. Original vertex‐wise area in FreeSurfer is defined as the sum of 1/3 the area of each adjacent triangular face on the FreeSurfer “white” surface (refined gray/white matter boundary estimate). That single‐vertex sum is not exactly constant across vertices because of slight non‐uniformities in the final relaxed state of the surface tessellation. However, the sum of vertex‐wise areas over a connected region of vertices exactly represents the summed original area of the enclosed triangles (plus the 1/3 fraction of triangles associated with the boundary vertices; along a straight edge of vertices, this last contribution corresponds to half of the area of the triangles just beyond the edge). The minimum areal increment that can be measured is roughly the average original vertex‐wise area, which is ∼0.6 sq mm.

## RESULTS

We first discuss the results obtained for each experiment in the study individually, followed by the overlap analysis results of reading activation with topological visual, auditory and somatomotor maps. A separate section is included to discuss several new and previously unreported sensory maps observed during the mapping experiments.

For the result figures corresponding to individual experiment results, we illustrate the amplitude of the vertex wise response for both reading experiment and phase‐encoded maps (ignoring phase) for a stairstep of *t*‐values. This is followed by figures illustrating single modality phase hue maps for sensory‐motor maps where hue is used to indicate the map coordinates.

The overlap figures use transparent overlays to indicate reading activations over single modality phase hue maps, finally leading up to a summary outline figure containing all four kinds of data. The language contrast used for overlap analysis is English vs. Hindi. Brain activation observed for English vs. Hindi contrast partially overlapped with retinotopic, tonotopic and somatomotor maps. For clarity, in overlap figures, only positive activation after thresholding and cluster correction is shown. The overlap results for different modalities are illustrated for several individual subjects and then for the group as a whole. For the cross‐subject average, the sensory‐motor maps are illustrated for two separate vertex thresholds; *P* < 0.05 (lower threshold) and *P* < 0.01 (higher threshold), corrected for multiple comparisons using cluster thresholding at *P* < 0.05. Because the phase‐encoded analysis effectively spreads the same amount of imaging data over a larger number of different effective conditions (e.g., different polar angles, sound frequencies, body parts) than the Reading experiment does, the regression analysis carried out on reading data will have more power. Hence the Reading data illustrated here uses a vertex threshold of *P* < 0.01 in all cross‐average images. For the individual subjects, results are illustrated at a higher vertex threshold of *P* < 0.001, corrected to *P* < 0.01 for both reading and mapping data due to the higher amount of noise present in single subject data.

The individual subject data are illustrated here to show that in general the pattern of activity was similar to the cross‐subject average. The majority of subjects showed reading activation patterns similar to subject‐1 and subject‐2, while subject‐3 had more restrained reading activation. All three of the individual subjects illustrated here had superlative comprehension task performance (vivid post‐scan explanations correctly citing most of the text concepts presented to them during the task); all were in their early 20's. Subject‐1 was scanned using EPI sequence while subjects 2 and 3 were scanned using multiband sequence.

Among the 20 subjects who took part in reading experiment, data from 3 subjects were excluded from the analysis owing to unsatisfactory performance in the target detection task and/or assessment of poor comprehension following the session and/or excessive movement (>1 mm). The activation for the target detection regressor (button press regressor) was used as an extra quality check to decide whether the subject performed the task as per the instructions during each run. All our included subjects had comparable performance across conditions in the target detection task and motor activation for the target regressor. Among our 17 good subjects, three subjects were scanned using a single‐slice EPI protocol and the remaining 14 subjects were scanned using multiband EPI, which had more complete brain coverage as well as higher signal‐to‐noise than the single‐slice EPI data. However, the EPI protocol did not cover the entire brain, especially the anterior‐ventral most portions of the temporal/frontal lobe (more so since participant's head was tilted forward for visual experiments as explained in experimental set‐up). We did a further analysis of the reading data excluding the three single‐slice EPI subjects. This revealed reading activation that continued up to the temporal pole as well as more anterior frontal activation. Hence for the group results of the Reading task, we have used data from the 14 multi‐band subjects (N.B.: this region was more fully covered in the EPI auditory mapping experiments because of more standard head positioning). The original group result from the full 17 subjects is included as a Supporting Information Figure. Re‐analysis of mapping data did not reveal any further coverage, and group results for mapping data include data from EPI subjects. Among the 17 (of the same) subjects who took part in somatomotor experiment, data from two subjects were excluded owing to high stimulus correlated head motion during the task. No exclusions were made for retinotopic and tonotopic experiments.

### Target Detection Response

**Figure 3:**
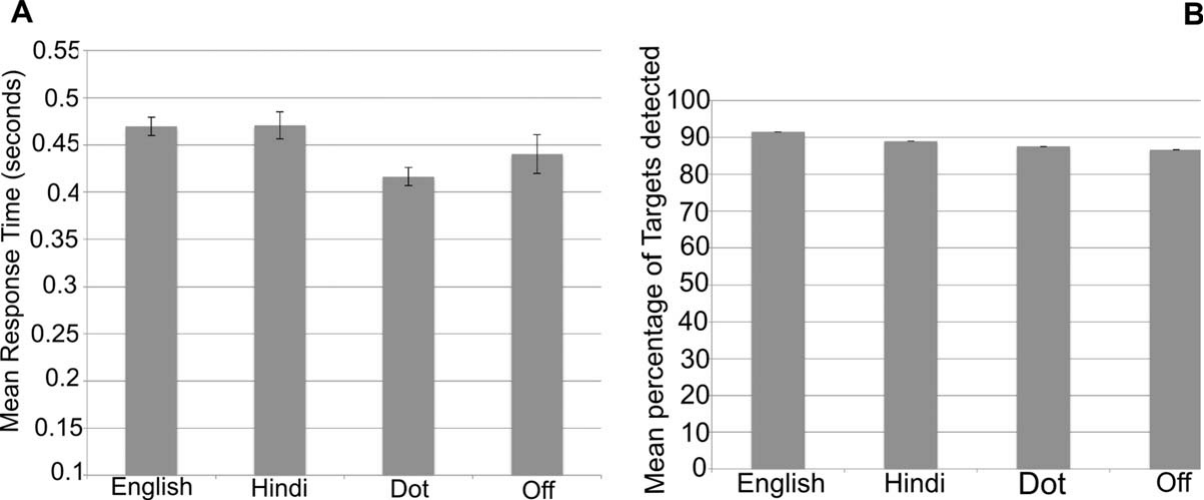
Target detection Performance results (Reading Experiment). Error bars represent one standard error from the mean.

Figure 3 shows the target detection response results based on the performance of the included subjects. The average response time for the group when the target occurred in English, Hindi, Dot and Fixation conditions are depicted in Figure 3A. On average, participants took 0.47 seconds ± 0.01 (SEM) to respond to the target when it occurred in English/Hindi conditions and 0.42 seconds ± 0.01 (SEM) and 0.44 seconds ± 0.02 (SEM) when they occurred in Dot/Off conditions. A Wilcoxon matched‐pairs signed‐ranks test indicated no significant differences between “English” (median= 0.45 seconds) and “Hindi” (median = 0.46 seconds) (*Z* = −1.681, *P* = 0.098). The differences between “English” and “Dot” (median = 0.42 seconds) and “English” and “Off” (median = 0.40 seconds) conditions were found to be significant (*Z* = −2.96, *P* = 0.002 for English‐Dot and *Z* = −2.02, *P* = 0.043 for English‐Off).

Figure 3B depicts the average target detection success rate across all participants for different conditions. Across all runs, there were 20 targets for the English condition, 18 for the Hindi condition, 23 for the Dot and 7 for Off condition. All participants except one (see below) had comparable detection performance irrespective of the condition in which the target occurred. One participant did not respond to all seven targets that fell on the fixation, though she consistently performed in all other conditions. All other quality measures were satisfactory for this subject and her data suggested that she did fixate during Off condition (the fovea had no activation in English vs. Fixation). We have therefore conservatively considered her data as valid. On average, the mean success rate for detecting targets when they occurred in English, Hindi, Dot and Off were 91.6%, 89.1%, 87.5% and 86.6% respectively. The average for Off condition is less mainly because of one outlier, the above mentioned subject. Excluding her data, the average success rate for Off condition was 92%. A Wilcoxon matched‐pairs signed‐ranks test was carried out to assess statistical significance of the average success rate. The median success rate for English, Hindi, Dot and Off were 90%, 89%, 87% and 100% respectively. There were no significant differences between English and any of the other conditions (English‐Hindi: *Z* = −1.733, *P* = 0.087; English‐Dot: *Z* = −1.949, *P* = 0.051; English‐Off: *Z* = −0.94, *P* = 0.94).

### Reading Activation

**Figure 4:**
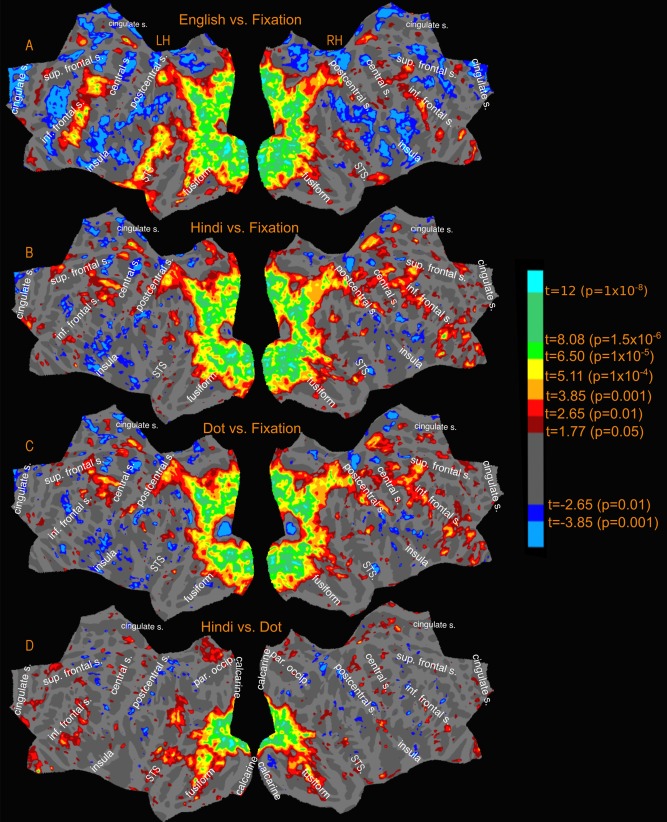
Reading Experiment‐Activation amplitude profile (uncorrected) for relevant contrasts.

**Figure 5:**
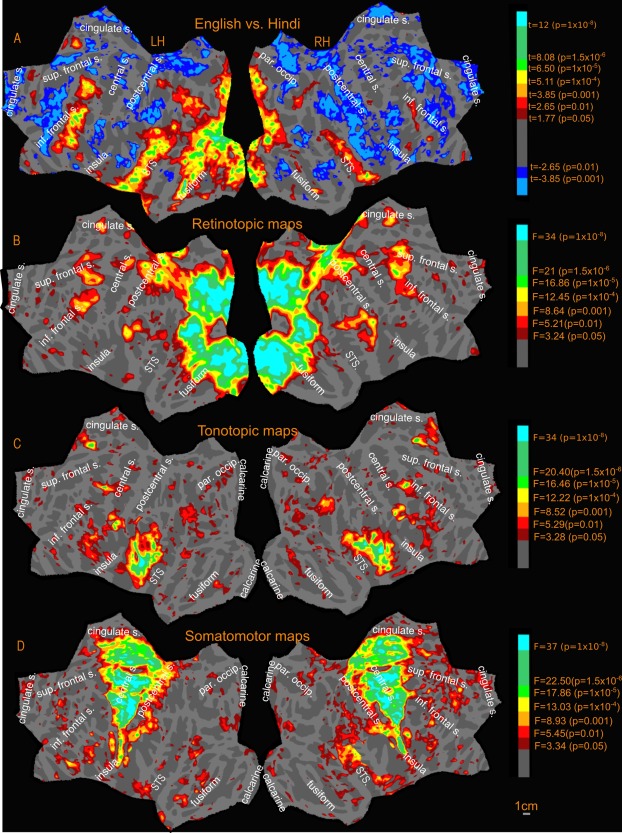
Activation amplitude profile (uncorrected) of Sensory‐motor maps and English vs. Hindi contrast.

Figure 4 and the top of Figure 5 illustrate the average cross‐subject activation for a stairstep of *t*‐values (all beyond a minimum threshold of *P* < 0.05, uncorrected) for each condition (English, Hindi and Dot) relative to fixation (Fig. 4A–C), and those for two main contrasts, Hindi vs. Dot (Fig. 4D), and English vs. Hindi (Fig. 5A). The reading activation illustrated in the group results come from 14 subjects who were scanned using the whole brain multiband sequence. Supporting Information Figure S1 shows the cross‐subject results of all 17 subjects including the three subjects who were scanned using the single‐slice EPI sequence. The main contrast used to assess reading comprehension is English vs. Hindi. In the later figures depicting overlap with sensory‐motor maps, transparent white regions outlined in black depict the regions that showed significantly higher activation when reading English compared to Hindi. In all subjects, activation for English > Hindi was more widespread and pronounced in left hemisphere than in right hemisphere, as expected. The right hemisphere regions activated were a mirror image subset of the left hemisphere counterparts.

The cross‐subject reading activation for English vs. Hindi (Fig. 5A) is spread around three main connected regions in the left hemisphere. The first region was anterior to the occipital pole, extending laterally into the occipital cortex with two “offshoots.” The first offshoot stretched across the intraparietal sulcus, covering regions in the inferior and superior parietal lobules. The second offshoot stretched along inferior temporal gyrus and occipito‐temporal sulcus and extended ventrally onto the fusiform gyrus joining the medial activation that extended from calcarine fissure to the fusiform gyrus. Less extensive but prominent activation was observed in medial regions in the cuneus, precuneus and at the isthmus of the cingulate gyrus. For English, Hindi, and Dot (versus Fixation), roughly the same regions in the occipital and parieral lobe were activated. However, the activation differences were most significant in those regions for English vs. Hindi. The activation along occipito‐temporal sulcus and fusiform gyrus was also mainly observed in the English condition.

The second main region (English vs. Hindi) is the superior temporal cortex, covering regions along the superior temporal gyrus and sulcus extending into the middle temporal gyrus and supramarginal gyrus. Except for a small region in posterior STS, the Hindi and Dot conditions do not have any significant temporal lobe activation.

The third region included two distinct frontal regions—one near the precentral sulcus and another more extensive region near the inferior frontal sulcus in the pars opercularis and pars triangularis region. The right hemisphere activation profile is similar but covering a much smaller total extent of cortical area. The activation near the precentral sulcus is present for Hindi and Dot conditions as well, with no significant differences in the Hindi vs. Dot contrast (Fig. 4D). The activation observed for English in the inferior frontal region near pars opercularis is absent in both Hindi and Dot conditions.

The reading activations of subject‐1 and subject‐2 were strikingly similar to the cross‐subject profile, with activated regions corresponding to the three main regions just described in the left hemisphere. Individual subject‐3 (see below) showed the greatest variation from the average, with more extensive activation observed in superior temporal sulcus and middle temporal gyrus, but then less extensive activation elsewhere in the cortex.

### Retinotopic Maps

**Figure 6:**
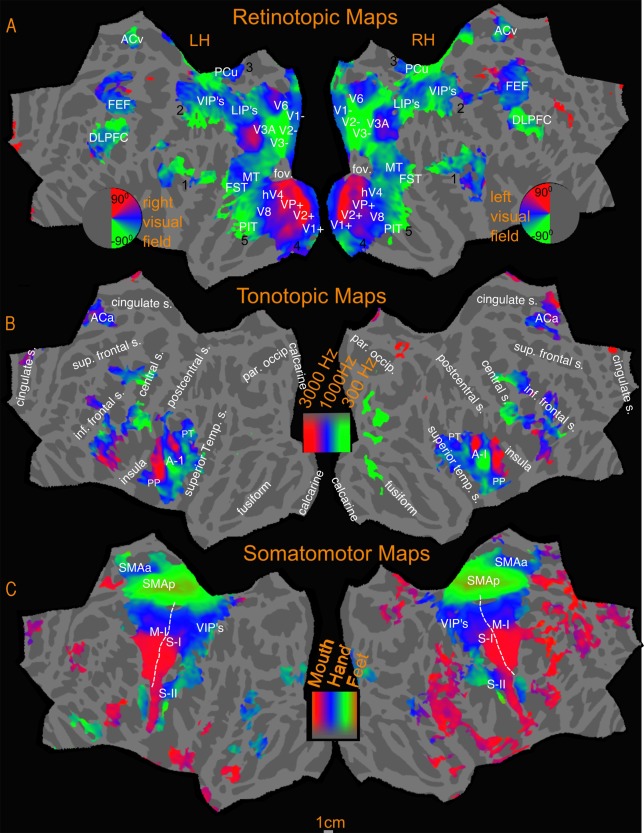
Sensory‐motor maps‐ Polar phase maps. Activations are illustrated at *P* < 0.05, corrected to *P* < 0.05.

**Figure 7:**
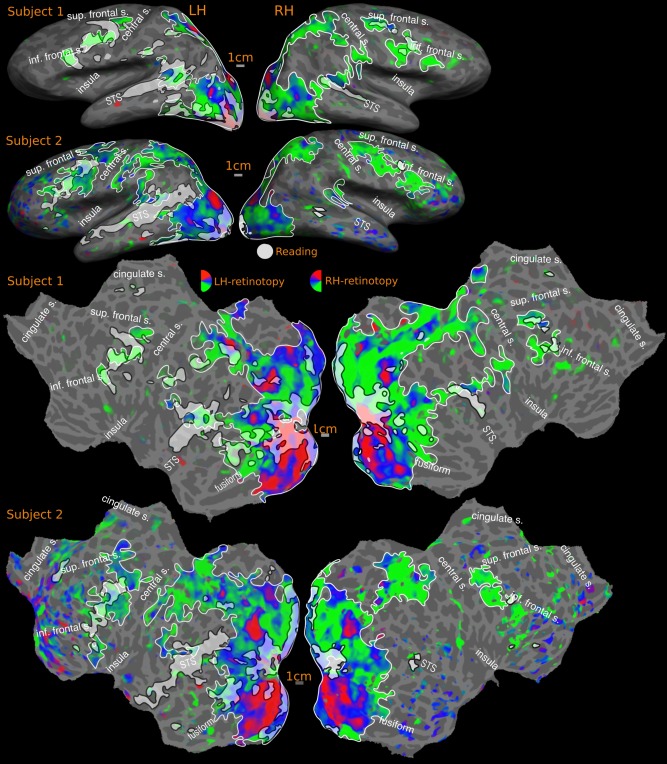
Overlap of reading (English vs. Hindi) with RETINOTOPIC maps‐ individual subjects. Activations are illustrated at *P* < 0.001, corrected to *P* < 0.01. Percentage of reading activation overlapping with retinotopy: Subject‐1: 58% (LH), 83% (RH); Subject‐2: 48% (LH), 81% (RH). In all overlap figures (Figs. 7–12), sensory‐motor maps are represented using red, blue and green colors, with map borders outlined in white. Reading activation uses a uniform white color with borders indicated using black.

**Figure 8:**
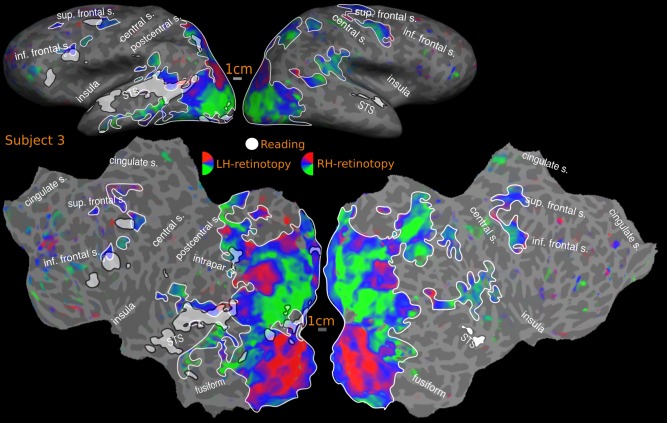
Overlap of reading (English vs. Hindi) with retinotopic maps‐ individual SUBJECT. Activations are illustrated at *P* < 0.001, corrected to *P* < 0.01. Percentage of reading activation overlapping with retinotopy: Subject‐3: 28% (LH), Nil (RH)

**Figure 9:**
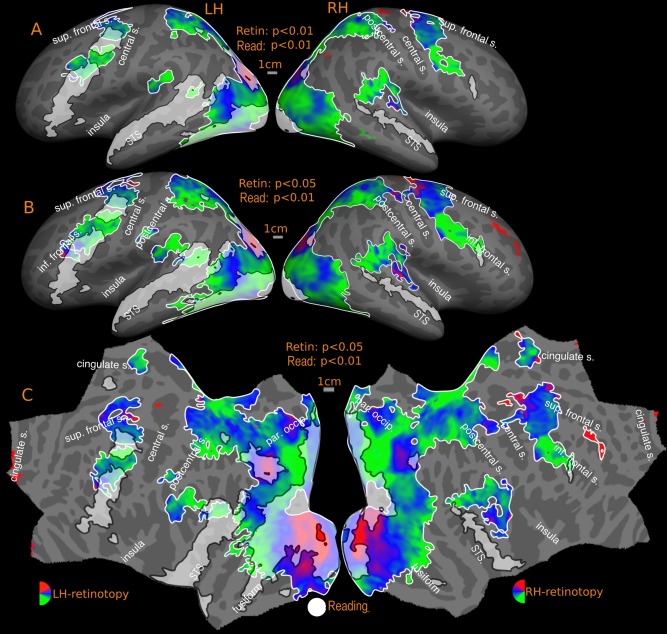
Overlap of reading (English vs. Hindi) with retinotopic maps‐ GROUP. Reported *P* values are vertex thresholds used before cluster thresholding. All activations are cluster thresholded to *P* < 0.05. Reading activation is illustrated at *P* < 0.01 (vertex threshold) in A and B. Retinotopic activation is illustrated at two different vertex thresholds‐ *P* < 0.01 (A) and *P* < 0.05 (B). C represents the flattened version of B. Percentage of reading activation overlapping with retinotopy‐ A: 64% (LH), 75% (RH); B: 69% (LH), 77% (RH).

The retinotopic maps (amplitude in Fig. 5B, phase in Fig. 6A; see also phase underlays in Figs. 7, 8, 9) branch out anteriorly from the occipital pole into several “streams” (numbered 1–5 in black in Fig. 6A). In all phase maps, lower visual field is green, horizontal meridian is blue, and upper visual field is red (all contralateral). One stream extends through area MT into the superior temporal sulcus and reaches the posterior lateral sulcus (leaving a few disconnected regions in between, which join up as threshold is slightly lowered). A second stream stretches along the intraparietal sulcus and arrives at the superior part of the postcentral sulcus. A third stream spreads across the parieto‐occipital sulcus (POS) into the medial posterior parietal cortex and precuneus, ending at the cingulate sulcus visual area [Huang and Sereno, 2013]. A fourth stream runs across the POS into retrosplenial cortex at the isthmus of cingulate gyrus (iCG) and continues to the edge of cortex just under the splenium of the corpus callosum. Finally, a fifth stream follows the collateral sulcus and fusiform gyrus into the ventral occipitotemporal lobe. A further disconnected set of retinotopic maps, including the frontal eye fields (FEF), frontal poly‐sensory zone [Huang et al., 2012], and dorsolateral prefrontal cortex (DLPFC) are found in the frontal cortex. These maps are similar to those reported previously in Huang and Sereno [2013] using a similar stimulus. We also found a previously unreported visual map in the anterior cingulate region, referred to in Figure 6A as anterior cingulate visual area (ACv), which corresponds to the human dorsomedial eye‐fields (discussed further under New maps).

The mapped regions in both lower and higher‐level visual areas unanimously preferred contralateral visual stimulation. The retinotopic activation pattern in individual subjects (Figs. 7 and 8) closely follows the average pattern in Figure 6A. It should be noted that the lack of retinotopy in the result figures around the fovea is an artifact expected with central fixation (no periodic signal change will be present since the subject is fixating at all times); these regions are known to be retinotopic [Schira et al., 2009] and are considered so for overlap analysis.

### Tonotopic Maps

**Figure 10:**
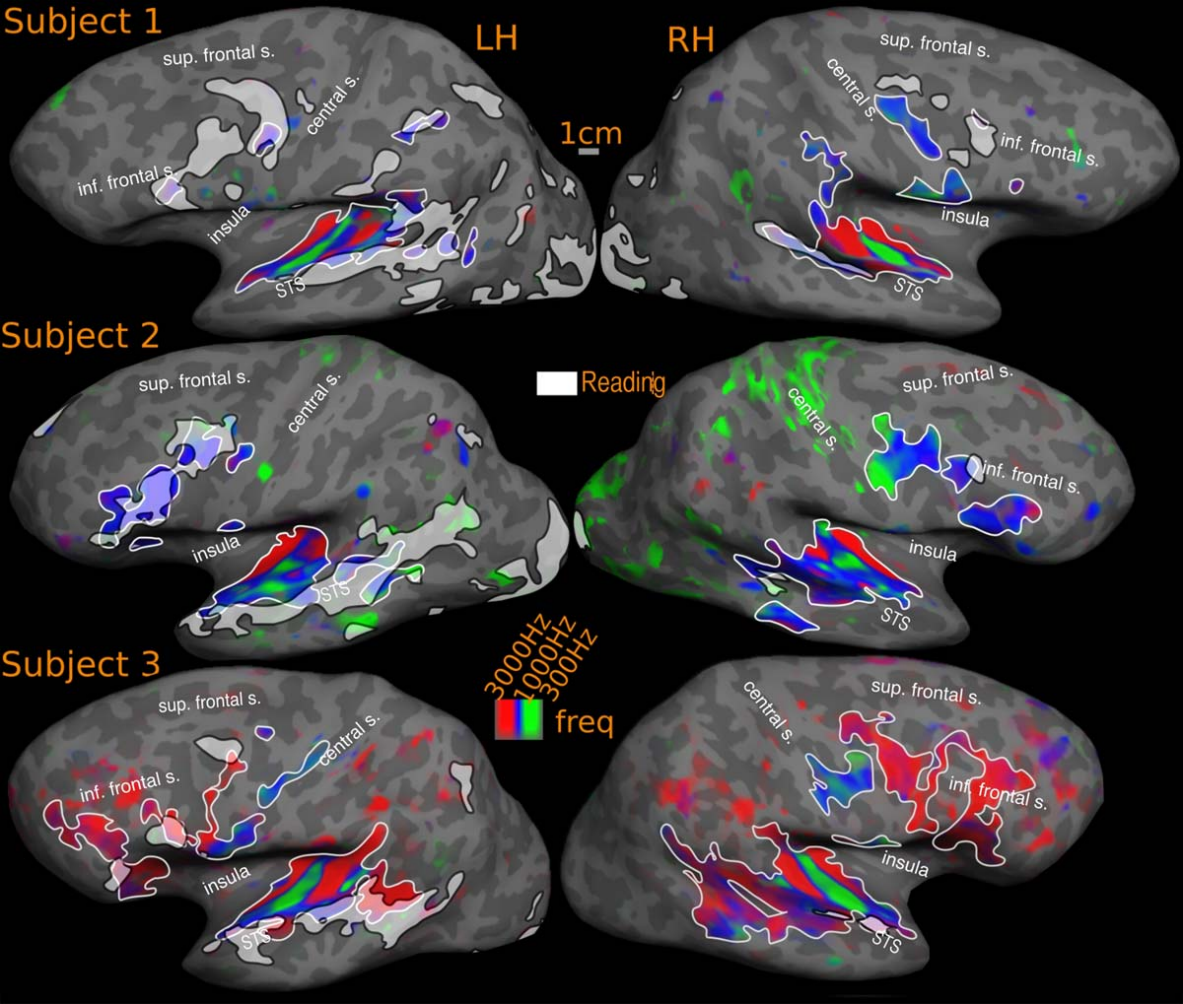
Overlap of reading (English vs. Hindi) with tonotopic maps‐ individual subjects. Activations are illustrated at *P* < 0.001, corrected to *P* < 0.01. Percentage of reading activation overlapping with tonotopy: Subject‐1: 6% (LH), 10% (RH); Subject‐2: 14% (LH), 7% (RH); Subject‐3: 32% (LH), 54% (RH)

**Figure 11:**
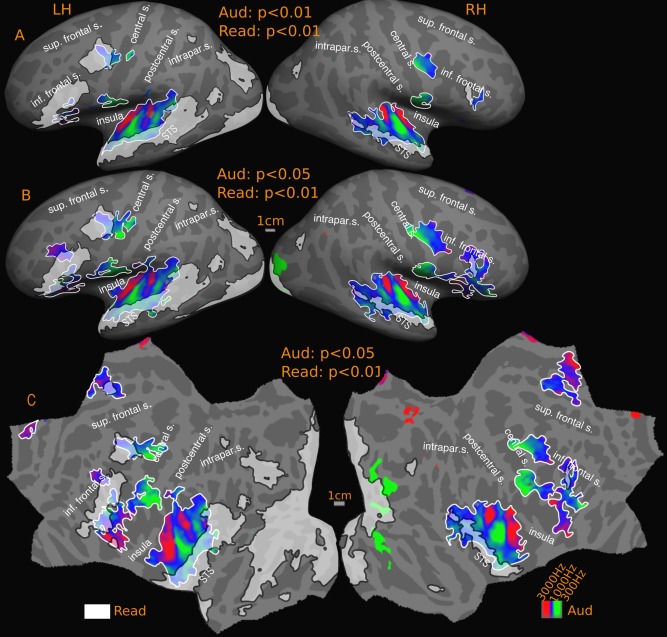
Overlap of reading (English vs. Hindi) with tonotopic maps‐group. Reported *P* values are vertex thresholds used before cluster thresholding. All activations are cluster thresholded to *P* < 0.05. Reading activation is illustrated at *P* < 0.01 (vertex threshold) in A and B. Tonotopic activation is illustrated at two different vertex thresholds‐ *P* < 0.01 (A) and *P* < 0.05 (B). C represents the flattened version of B. Percentage of reading activation overlapping with tonotopy‐ A: 7% (LH), 14% (RH); B: 11% (LH), 20% (RH).

Tonotopy is observed in several regions in the cortex (amplitude in Fig. 5C, phase in Fig. 6B; see also phase underlays in Figs. 10 and 11). In all phase maps, lower frequencies are green, middle frequencies blue, and high frequencies red. The tonotopic maps in and around primary auditory cortex in the lateral fissure are well known and followed characteristic features previously reported in the literature [Formisano et al., 2003; Talavage et al., 2000, 2004]. In both the group maps and the individual subject maps, there is a high‐to‐low‐to‐high frequency progression across Heschl's gyrus (HG), moving diagonally (medially and anteriorly) across the temporal plane. This region is flanked by higher frequency representation anteromedially toward the planum polare (PP) and posteromedially toward the planum temporale (PT). Posterior to HG on the lateral PT on the superior temporal gyrus, an additional low frequency focus, and two small high frequency foci are also observed. A few subjects also exhibited tonotopic responses extending inferiorly beyond the fundus of the superior temporal gyrus onto the middle temporal gyrus (e.g. subject‐3).

Tonotopic maps were also found in several frontal regions (discussed further under New maps). In the cross subject average those regions are most significant in inferior frontal cortex, and on the precentral gyrus extending posteriorly into the central sulcus and anteriorly into the precentral sulcus, reaching the inferior/anterior end of the precentral sulcus. While tonotopy in human frontal lobe has received scant attention so far, our results suggest that consistent frontal tonotopy is observed with only a moderate degree of variation across subjects.

Finally, a distinct tonotopic region was found in both hemispheres in the anterior cingulate region (ACa in Fig. 6B, discussed further under New maps). The anterior cingulate tonotopic region is immediately anterior to the anterior cingulate visual maps (ACv) described above, and does not overlap it.

### Somatomotor Maps

**Figure 12:**
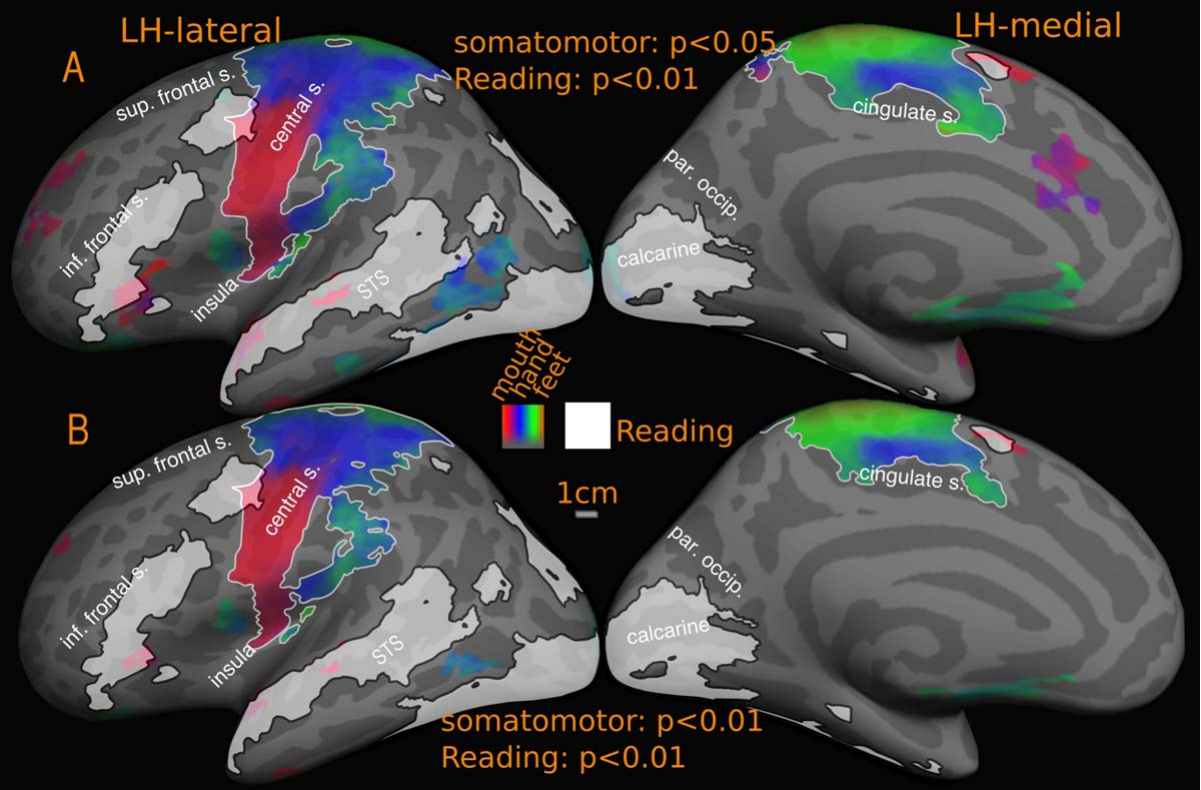
Overlap of reading (English vs. Hindi) with somatomotor maps‐group. Reported *P* values are vertex thresholds used before cluster thresholding. All activations are cluster thresholded to *P* < 0.05. Reading activation is illustrated at *P* < 0.01 (vertex threshold) in A and B. Somatomotor activation is illustrated at two different vertex thresholds‐ *P* < 0.05 (A) and *P* < 0.01 (B). Percentage of reading activation overlapping with somatomotor maps‐ A: 0.01% (LH), Nil (RH); B: 0.01% (LH), Nil (RH).

Figure 12 illustrates the group level somatomotor maps (noise corrected using ICA‐AROMA) on the inflated surface (also see amplitude, Fig. 5D, and phase maps, Fig. 6C, for a flattened view) overlapped with reading (English vs. Hindi) activation. The somatomotor mapping revealed a highly significant ventral‐to‐dorsal face‐to‐leg somatotopic representation in M‐I proper and premotor cortex, as well as in a number of post‐central areas including primary somatosensory cortex (including areas 3a, 3b, 1, and 2), parts of area 5, two small maps in the upper bank of the lateral sulcus (S‐II and related areas), some somatotopy extending superiorly out of the lateral sulcus (7b), and most of VIP+, where we identified a characteristically medially displaced face representation in the postcentral sulcus (red), as well as a more medial lower‐visual‐field‐overlapping lower body representation recently revealed in Huang et al.'s. [2012] mapping study that used an air puff body suit. Finally, we activated two additional smaller body representations (leg‐to‐face and face‐to‐leg) on the medial surface moving posterior to anterior in supplementary motor area (SMA) at both group and individual level. Though neither of these appears to reach all the way to the face (red) in the average, the reason for this is that inter‐subject variation in the exact location of these small maps resulted in flattening the phase range in the average; a full face representation was visible in a number of other individual subjects (data not shown). During mapping of the face, participants softly mouthed syllables, which they were nevertheless able to hear, and which are similar to speech sounds, which we believe explains some face‐correlated activation observed in temporal auditory areas. In order to assess the possible auditory contribution, we analyzed the activation due to auditory cue, (by doing a Fourier analysis at the frequency at which auditory cue occurred, 88 cycles/scan and thresholded at *P* < 0.05), and masked out the activated regions. These masked regions mainly involved the auditory cortex and small regions in the insula, frontal cortex and medial anterior cingulate.

The non‐ICA corrected group result is shown in Supporting Information Figure S2. As can be seen, there is larger face‐correlated activation in anterior frontal areas and the anterior temporal lobe. This was likely caused by B0 deformations due to changing the distribution of air in the oral cavity during active mouth movements (these brain regions are closest to the oral cavity). There was no map‐like structure (phase spread or reversals) in the apparent activity in these regions, nor any overlap with reading activation. By contrast, the region on the anterior bank of the central sulcus near the border between the face and hand representations of M‐I—which is substantially further away from the oral cavity—was a poly‐sensory zone activated also by passive visual and auditory stimulation, as can be seen in individual maps and group subject maps (Fig. 6).

### Overlap of reading (English vs. Hindi) activation with visual, auditory, and somatomotor maps

**Figure 13:**
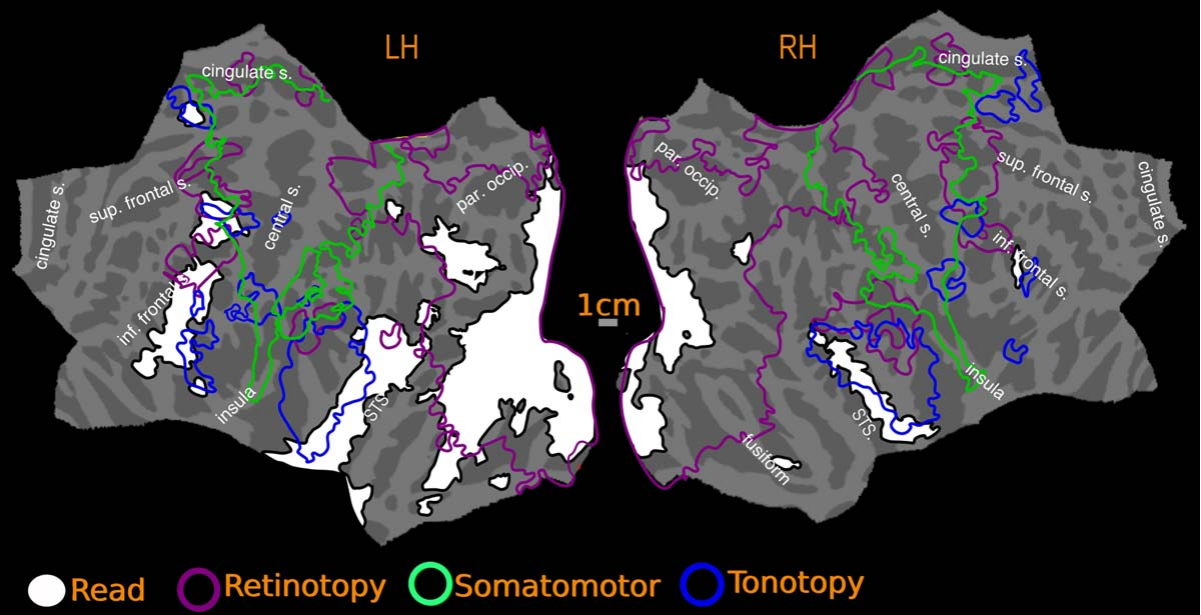
Overlap of reading (English vs. Hindi) with all maps‐group. Reading activation and maps are illustrated at vertex threshold *P* < 0.01, cluster corrected to *P* < 0.05

Figures 7, 8, 9, 10, 11, 12, 13 show the overlap of reading (English vs. Hindi) activation with topological visual, auditory and somatomotor maps. For the cross‐subject activations, the visual, auditory, and somatomotor activations were assessed for two different hard vertexwise thresholds before cluster exclusion correction: *P* < 0.01 (higher threshold) and *P* < 0.05 (lower threshold). Reading activation uses a single higher hard vertex threshold of *P* < 0.01. For single subject activations, reading, retinotopic, and tonontopic maps are hard thresholded at *P* < 0.001, corrected to *P* < 0.01. The overlap estimates below, are expressed as the percentage of reading activation intersecting with sensory‐motor maps. The quantitative results include an overall estimate, where the percentage of total reading activation overlapping with retinotopic, tonotopic and somatomotor maps is reported for each hemisphere. Additionally, each region (frontal, temporal and occipito‐parietal) for reading activation is considered separately, and corresponding overlap is expressed as a percentage of the regional reading activation. The quantitative estimates are summarized in Table 1.

**Table 1:** Overlap of reading activation with retinotopic and tonotopic maps

			Group				
			Higher threshold	Lower threshold	Subject‐1	Subject‐2	Subject‐3
Retinotopic maps (as a percentage of reading activation)	Left hemisphere	Overall	64%	69%	58%	48%	28%
		Frontal	27%	40%	34%	40%	11%
		Temporal	6%	17%	17%	5%	13%
		Occipital	94.1%	94.3%	100%	100%	100%
	Right hemisphere	Overall	75%	77%	83%	81%	Nil
		Frontal	Nil	53%	69%	38%	Nil
		Temporal	Nil	1%	18%	Nil	Nil
		Occipital	98%	98%	100%	100%	Nil
Tonotopic maps (as a percentage of reading activation)	Left hemisphere	Overall	7%	11%	6%	14%	32%
		Frontal	14%	31%	8%	51%	44%
		Temporal	17%	24%	17%	23%	39%
		Occipital	Nil	Nil	Nil	Nil	Nil
	Right hemisphere	Overall	14%	20%	10%	7%	54%
		Frontal	13%	93%	8%	33%	Nil
		Temporal	49%	59%	71%	29%	61%
		Occipital	Nil	Nil	Nil	Nil	Nil

#### Reading overlap with retinotopic maps

The retinotopy/reading overlap for the cross‐subject surface average is shown in Figure 9. The reading activation in the occipital and parietal cortex in both hemispheres mostly fall within retinotopically mapped regions. In the left hemisphere, the overlapping regions include early visual areas such as V1, V2, ventral V3, extending to posterior inferior temporal cortex all the way to the lateral surface reaching MT and immediately surrounding areas, after which the lateral activation is attenuated. There is a more discontinuous overlap on the superior bank of the calcarine sulcus, with V1 and V2 activated, but little activation in dorsal V3. There is a strong activation again in the vicinity of V3A and immediately anterior areas. The retinotopic branch extending across MT overlaps with the reading activation in the posterior STS. The rest of the reading activation in the STS is outside the bounds of any retinotopy. In the frontal cortex, there is substantial overlap with both maps in the FEF region as well as more laterally located DLPFC. In the right hemisphere, reading activation in occipital cortex is largely limited to early visual areas V1 and V2. On the lateral occipital side, significant activation is observed immediately anterior to V3A. The STS activation overlaps partially with retinotopic maps in the posterior STS regions, and the right frontal activation overlaps partially with retinotopy in the frontal cortex. The reading activation around the fovea region is considered as overlapping with retinotopy, as the lack of retinotopy in this region is an artifact expected with central fixation (no periodic signal change will be present since the subject is fixating at all times); however, these regions are known to be retinotopic [Schira et al., 2009] and are considered so for overlap analysis.

With regards to quantitative overlap estimate, over the entire cortex, at the higher threshold, more than 60% of the cross‐subject reading activation in both hemispheres falls within retinotopic areas (LH: 64%, RH: 75%); the overall overlap rises to 69% in the left hemisphere and 77% in the right hemisphere at the lower threshold (see Fig. 9). We previously described three main regions of reading activation—occipito‐parietal, superior temporal and frontal. The reading activation in region‐1, which includes the occipital lobe, inferior temporal lobe, and superior parietal lobe falls mostly within retinotopic regions in both hemispheres (LH: 94% and RH: 98%, for high and low thresholds). In reading region‐2, the superior temporal cortex, in the left hemisphere, the overlaps were 6% (higher threshold) and 17% (lower threshold). In the right hemisphere, there is no overlap at higher threshold and a small 1% overlap at lower threshold. For reading region‐3, frontal cortex, which includes precentral and inferior frontal cortex, there is a 27% overlap with retinotopy at the higher threshold in the left hemisphere, which rises to 40% at the lower retinotopy threshold. In the right hemisphere, the frontal retinotopic maps lie just outside the borders of right hemisphere reading activation in frontal cortex at the higher threshold; at the lower retinotopy threshold, they extend to cover almost 53% of the right hemisphere frontal reading activation.

For two individual subjects subject‐1 and subject‐2 (Fig. 7), the overall left hemisphere retinotopy/reading overlap is 53% (subject‐1: 58% and subject‐2: 48%), while the right hemisphere overlap is above 80% (subject‐1: 83% and subject‐2: 81%). Zooming in on individual regions, as with the cross‐subject average, there is almost 100% overlap between reading and retinotopy in occipito‐parietal cortex in both hemispheres. In the left hemisphere frontal and temporal reading areas, overlap with retinotopy is 34% and 17% in subject‐1 and 40% and 5% in subject‐2. The corresponding overlaps for the right hemisphere of these subjects are 69% (frontal) and 18% (temporal) for subject‐1, and 38% (frontal) and 0% (temporal) for subject‐2.

In subject‐3 (Fig. 8), the frontal and temporal reading profile varies considerably and has a lower overlap (LH: 28%, RH: Nil) with retinotopic maps than it does with tonotopic maps (see below). The individual left hemisphere overlap figures in frontal and temporal cortex are 11% and 13%. There is no overlap in the right hemisphere.

The retinotopic map underlays illustrated in Figures 7, 8, 9 use the same color scale as the average (lower contralateral field green, horizontal meridian blue, upper field red). In the group results we found that in both hemispheres, the occipital reading activation overlaps roughly equally with lower, middle, and upper visual fields. In higher‐level retinotopic areas, there was a slight predominance of horizontal meridian and lower visual fields. There are two reasons for this. First, because retinotopic maps in higher‐level visual areas are smaller, slight inter‐subject displacements in the location of these maps has a tendency to reduce the range of polar angles in the average map. This 'regression toward the horizontal meridian' results in an overrepresentation of the horizontal meridian in the average. Another possible source of a horizontal meridian and particularly lower field emphasis is that subjects in our direct‐view experiments had to lower their gaze somewhat to fixate the center of the close‐up screen. Given that there is evidence for head‐centered remapping of receptive fields in some higher visual areas [see Sereno and Huang, 2006], the lowered gaze may have remapped the entire visual field slightly toward the lower field in these higher‐level areas, resulting in an overemphasis of the lower field in both individual subjects as well as the average.

#### Reading overlap with tonotopic maps

The most obvious overlap is in temporal cortex, where the reading task consistently activates an anterior‐posteriorly elongated region in superior temporal cortex. In the left hemisphere average, the tonotopic maps (Fig. 11, and compare Fig. 5A,C) overlap reading activation on the superior temporal gyrus and part of the upper bank of the STS. The inferior edge of the reading activation in turn overlaps the superior edge of middle temporal visual areas. Most subjects (and the average) showed more reading overlap with retinotopic maps than tonotopic maps. However, this was reversed in some subjects (e.g. subject‐3). In the right hemisphere average, the smaller superior temporal reading activation partially overlaps tonotopy.

The frontal lobe reading activation also overlaps partially with frontal tonotopic maps. There is a significant overlap near the precentral sulcus, a potential site of multi‐sensory integration since this region contain both retinotopic and tonotopic maps. Additionally tonotopic maps also has significant overlap with the inferior frontal reading activation and there is a small but strong reading activation in anterior cingulate, which overlaps almost completely with the tonotopic map in the region. These (ACC and inferior frontal) regions are only activated while reading English. These findings are consistent in the cross‐subject average as well as in individuals.

The overall tonotopy/reading overlap (Fig. 11) estimate is 7% (LH) and 14% (RH) for the higher threshold, and 11% (LH) and 20% (RH) for the lower threshold in the cross‐subject average. In left temporal cortex, the level of overlap is 17% at the higher threshold and 24% at the lower threshold. In the right hemisphere, more of the temporal reading activation falls within tonotopy (49% at higher threshold, 59% at lower threshold). The tonotopic maps also overlap with frontal reading activation in both hemispheres: 14% (high threshold) and 31% (low threshold) in left hemisphere and 13% (high threshold) and 93% (low threshold) in the right hemisphere.

Our typical individual subjects (Fig. 10) exhibit a similar profile (overall overlap: subject‐1: LH: 6%, RH: 10%; subject‐2: LH: 14%, RH: 7%). In subject‐1, 8% of frontal and 17% of temporal lobe activation in left hemisphere overlaps with tonotopy. The corresponding figures for right hemisphere are 8% frontal and 71% temporal. For subject‐2, the left hemisphere overlap is 51% frontal and 23% temporal and for the right hemisphere, 33% frontal and 29% temporal. Subject‐3 has especially extensive tonotopic maps and shows a higher degree of overall overlap with tonotopy than with retinotopic areas (overall: 32% in left hemisphere and 54% in right hemisphere). Around 44% of frontal and 39% of temporal lobe left hemisphere activation falls within tonotopically defined regions. In the right hemisphere, nearly 61% of temporal reading activation overlaps with tonotopy. No significant frontal reading activation is observed for subject‐3 in the right hemisphere; and there are tonotopic maps in this region.

In the group results, the overlap between reading and tonotopy in the temporal cortex in both hemipsheres fall within the low to middle frequency range, avoiding high frequency regions in the lateral sulcus. The situation in frontal cortex is more mixed. In the left frontal cortex, the overlapping region lying between precentral and central sulcus is dominated by mid‐low frequencies closer to central sulcus and by high‐mid frequencies closer to the precentral sulcus. In the inferior frontal region, there are two separate overlaps, the more dorsal overlap region is predominantly high‐mid range frequencies while the more ventral region is predominantly mid range frequencies. In the right hemisphere, the overlap is only with the pars opercularis activation, where the tonotopic maps overlapping with reading has a high‐mid frequency distribution.

#### Reading overlap with somatomotor maps

In the cross‐subject average, there is only one overlap zone, near the face/hand boundary bordering the central sulcus in M‐I (Fig. 12). The overlapping region corresponds to 0.01% of total reading activation at higher/lower threshold. This constitutes 4% (high threshold) of the left frontal reading activation, rising to 5% at low threshold. There was no overlap in right hemisphere or SMA.

#### New retinotopic and tonotopic maps

The topological mapping experiments uncovered several sensory maps previously unreported in the literature. In non‐human primates, it has long been known that the anterior cingulate SMAs are directly adjoined by the dorsomedial FEF in primates [Purcell et al., 2012; Schiller and Chou, 1998]. So far, there have not been any reports on topological maps in a similar region in humans. Our data may provide the first observation of the topological organization of the human equivalent of the dorsomedial FEF (ACv in Fig. 6A), which possess a retinotopic map of the contralateral visual field (their location is more inferior than in non‐human primates, in line with similar superior‐toward‐medial‐wall movements of parietal retinotopic areas in humans comparable to LIP). While retinotopy in this region is reported here for the first time, there have been a number of reports of activation in this region associated with visuo‐spatial attention and eye movements [Jamadar et al., 2013; McDowell et al., 2008; Mesulam et al., 2001; O'Reilly et al., 2013; Pierrot‐Deseilligny et al., 2004]. In the reading experiment, all three conditions that involved eye movements—English, Hindi and Dot have significant activation overlapping the anterior cingulate retinotopic region. This activation disappeared in the English‐Hindi and Hindi‐Dot contrasts suggesting the activation is more relevant for eye movements or/and spatial attention than for comprehension per se.

Adjacent to the dorsomedial retinotopic map, we also found a previously unreported tonotopic map, here after referred to as the dorsomedial frontal “ear” fields (ACa in Fig. 6B). The anterior cingulate retinotopic map and the tonotopic map are separated by the SMA map and do not overlap each other or SMA. There is strong reading activation aligned with the tonotopic map in this region and unlike the reading activation in the anterior cingulate retinotopic region, this activation is strongest while reading English, compared to both the other conditions. The relatively smaller activation observed in Hindi and Dot disappears in the Hindi‐Dot contrast, while English‐Hindi has a significant activation, aligned with the dorsomedial frontal “ear” fields (Figs. 4 and 5A). Although there are no previous reports of tonotopy in human ACC, the anterior cingulate region is known to receive significant projections from the auditory cortex [Barbas et al., 1999; Paus, 2001; Petrides and Pandya, 1988]. Tonotopy‐related organization in this region has been reported in an electrical stimulation study of bat cingulate cortex, where the elicited ultrasound vocalizations were organized in a tonotopic manner [Gooler and O'Neill, 1987].

Finally, we found several previously unreported tonotopic maps in frontal cortex. The frontal tonotopy partially overlaps with retinotopic maps in the region as well as the reading activation found in the frontal cortex. As in the case of ACC, there exist strong reciprocal connections between auditory cortex and prefrontal cortex (PFC). These connections are also suggested to be topographic in nature. In monkeys, the rostral and orbital PFC (areas 10, 12, 13, 46 rostral) are connected with rostral belt and parabelt auditory cortex, while the caudal prefrontal regions (areas 8a, 12, 46 caudal) receive connections from the caudal belt and parabelt auditory cortex [Plakke and Romanski, 2014; Romanski et al., 1999].

## DISCUSSION

The results presented here provide a comprehensive qualitative and quantitative assessment of where, and by how much, processes relevant to naturalistic reading overlap with topological (neighbor‐preserving) visual, auditory, and somatomotor maps using high‐resolution surface‐based fMRI across the entire cortex. Nearly 80 fMRI sessions were analyzed with a start‐to‐finish cortical‐surface‐based processing pipeline. This study combines recent advances in lower‐level sensory‐motor mapping with a higher‐level cognitive task (naturalistic reading with matched eye movements across conditions). There were two related objectives.

The first was to accurately localize regions of interest in naturalistic reading comprehension by determining their exact relation to low and high‐level topological visual, auditory, somatosensory and motor maps. Topological mapping is a time‐tested method for accurately defining the boundaries of cortical areas. The figures presented here are the first illustrations of the relative location of naturalistic reading comprehension activation and topological visual, auditory and somatomotor maps across the entire cortex in the same group of subjects. In the process, we discovered several previously unreported tonotopic maps in frontal cortex that partially overlapped frontal retinotopic maps. In anterior cingulate cortex, we found two additional new maps—a visual map and a tonotopic map, both near the SMA but not overlapping each other, or the SMA. A strong reading (English vs. Hindi) activation overlapped the left anterior cingulate tonotopic map. Our data sets benefitted from the latest multi‐band pulse sequences, allowing us to map the whole brain without losing temporal SNR, as well as from optimized sensory mapping stimuli, which together may account for why we were able to visualize those new maps.

Second, we wanted to provide a quantitative estimate for the level of overlap between activations observed during naturalistic reading comprehension and topological sensory‐motor maps, which are driven by relatively low‐level, sensory‐motor stimuli. Our finding is that more than 70% of reading activations (English vs. Hindi) in the left hemisphere and 89% in the right hemisphere fall within regions containing topological visual, auditory, somatosensory, or motor maps in the group average. At least 37% of the reading activation in frontal cortex, 23% in temporal cortex, and 94% in parietal and occipital cortex falls within visual, auditory or somatomotor maps. The overlap figures rise to nearly 60% in frontal cortex and 40% in temporal cortex if the threshold for detecting topological mapping is lowered to *P* < 0.05 (corrected). Given that smaller, higher‐level maps are more difficult to visualize than large, metabolically active, early visual areas, these figures serve as a conservative rather than an overly optimistic estimate [see Gonzalez‐Castillo et al., 2012]. We should also point out that the “overlap” defined here could only be measured at the resolution of our fMRI voxels. An overlap voxel might be identified if the two kinds of signals were within the same neuron, within distinct but adjacent neurons, within different 50 micron wide minicolumns, or within different ∼1mm wide columns (see Lund et al., 1993), all of which could be within the same voxel.

While occipital cortex and immediately surrounding regions have long been considered to be quintessentially visual, language research has often regarded activation elsewhere as falling outside the bounds of vision. For example, the classical language areas in superior temporal cortex and frontal cortex are often considered to be non‐visual. However, in the last decade or so, several additional retinotopic maps were discovered in frontal cortex [Hagler and Sereno, 2006; Hagler et al., 2007; Kastner et al., 2007], parietal cortex [Huk et al., 2002; Schluppeck et al., 2005; Sereno and Huang, 2006; Sereno et al., 2001, 2003; Silver et al., 2005; Silver and Kastner, 2009; Swisher et al., 2007] and temporal cortex [Huang and Sereno, 2013; Sereno et al., 2003]. The higher‐level retinotopic maps in frontal cortex were originally discovered using working memory and spatial attention paradigms [Hagler and Sereno, 2006; Kastner et al., 2007]. Those studies suggested that topological maps may serve as a convenient method of allocating working memory, or maintaining pointers to specific content, even (or more so!) for tasks that don't explicitly require attention to spatial location, but that require attention to content. The present study shows that reading activation in frontal cortex has significant overlap with these retinotopic maps.

In addition to confirming previous reports of retinotopic maps in frontal cortex, we also found new evidence for consistent tonotopy both in DLPFC and ventral PFC. While tonotopy in superior temporal cortex has been the subject of several studies [Da Costa et al., 2011; Dick et al., 2012; Formisano et al., 2003; Humphries et al., 2010; Langers and Van Dijk, 2012; Talavage et al., 2000, 2004] there is virtually no literature on tonotopy in human frontal cortex. There is, by contrast, a wealth of neuroimaging studies providing evidence that the human frontal lobe is active during general auditory tasks [Alain et al., 2001; Arnott et al., 2004; Gaab et al., 2003; Kiehl et al., 2001; Koelsch et al., 2009; Muller et al., 2001; Platel et al., 1997; Rämä et al., 2004]. Neurophysiological and neuroanatomical findings in non‐human primates show that both dorsolateral and ventral PFC is reciprocally interconnected with auditory regions in the temporal cortex [Hackett et al., 1999; Plakke and Romanski, 2014; Romanski et al., 1999]. The frontal reading activation overlaps the tonotopic maps in the region, perhaps for a similar reason as for the retinotopic map overlap (e.g., memory allocation using topological maps as pointer buffers).

Any activation of higher‐level visual and auditory areas during reading comprehension has often been dismissed with the claim that readable, understandable text (here English) attracts more attention than unreadable, incomprehensible text (here Hindi characters of the same word length, fixated with the same controlled pattern of saccades). An assumption prevalent in the literature is that comprehension and reading processes are dissociable from sustained attention, and that they can be dissociated by using a control task matched in attentional complexity. Recent research however suggests that attention is a cortex‐wide dynamic process whereby resources are flexibly allocated to the attended function as opposed to a simple mechanism that merely modulates baseline response level [Çukur et al., 2013, Peelen and Kastner, 2014]. But this also implies that subtractive analyses attempting to control for attention factors assuming a static modular nature of attention may be overly conservative. In the present study, any task‐related attention and working memory processes are considered relevant and necessary for naturalistic reading comprehension and have not been subtracted out. Low level visual processing including saccade preparation and execution (eye movements), and task‐irrelevant attention are controlled by subtracting out activation due to the combination of “Hindi” font, directed naturalistic saccades, and the target detection task. In addition to facilitating a naturalistic reading experience, the presentation of text by highlighting the next word drives the exogenous attentional network [Posner, 1980] and triggers a saccade toward the highlighted spatial location. While spatial attention can be modulated without saccades, saccades rarely occur without being directed by attention. Substantial evidence exists suggesting that an eventual saccade target peripheral to fixation attracts attention during saccadic planning [Melcherand Colby, 2008; Zhao, 2012], and that attention then quickly returns to the center of gaze after the saccade to the target has been made. Finally, the random color change detection task performed by the subjects during the experiment across all conditions served as a control for endogenous attention. In all three conditions‐ English, Hindi and Dot, significant activation was observed bilaterally near intra parietal/postcentral sulcus, FEF, and dorsomedial frontal eye fields, regions known to be activated by visuo‐spatial attention and eye saccades [Jamadar et al., 2013; McDowell et al., 2008; Müri and Nyffeler, 2008; O'Reilly et al., 2013; Pierrot‐Deseilligny et al., 2004]. The activations in dorsomedial frontal eye fields and parts of parietal cortex are fully attenuated in English vs. Hindi contrast (as well as in Hindi vs. Dot contrast, in fact Hindi and Dot has slightly better activations in these areas). In FEF, while the activation disappears in Hindi vs. Dot contrast, a good part of it is still highly significant in English vs. Hindi contrast, suggesting that the FEF may have a larger role in comprehension, an observation supported by other studies [Choi et al., 2014; Hasson et al., 2008].

However, not all frontal regions were activated for all three conditions. The more anterior activation near left inferior frontal sulcus/gyrus (a classical language region) overlapping partially with the DLPFC visual maps and tonotopic maps were unique to reading and comprehending English passages. Similarly the activation observed in left cingulate dorsomedial frontal 'ear' fields region is unique to the English condition. The reading activation in the left inferior frontal region partially overlaps with visual and tonotopic maps in the region, while the left cingulate reading activation falls entirely within anterior cingulate tonotopic maps.

As mentioned above, part of the activation observed could be due to sustained attention and working memory processes engaged in reading meaningful passages; but the functional dissociation of reading processes was very specifically not the goal of the current study. Reading in real situations is a computationally intense, attention‐ and working memory‐demanding contextual process that involves tying together information gleaned from linear symbol streams across seconds and minutes. The idea that these processes are recruited in language processing is not a new one [see Duncan et al., 2000; Fedorenko and Thompson‐Schill, 2014]. What is not clear though from the present literature is where the boundaries of these putative multi‐functional regions or classical frontal language regions lie. Although we cannot distinguish between domain general processes and purely linguistic processing from the contrast used here, the present study does define precisely where reading activation zones are relative to topological cortical maps in the frontal cortex. Dissociating linguistic and non‐linguistic elements in the frontal activation observed in the English vs. Hindi contrast is an important question for future research and we believe similar contrasts using non‐linguistic stimuli will help delineate functional specifications in this region, without downplaying comprehension specific processing taking place in these regions.

Despite being a silent reading task, there is also significant overlap with low‐level tonotopic maps in the superior temporal gyrus. It has been reported previously that tonotopic maps in auditory cortex overlap with temporal voice areas [Belin et al., 2000] in STG [Moerel et al., 2012]. Additionally, silent active reading has been shown to activate temporal voice areas [Perrone‐Bertolotti et al., 2012]. Our results are consistent with these findings. Our results also confirm a previous finding that the tonotopic maps overlapping reading activation in the STG have a bias toward the lower frequencies [Moerel et al., 2012], a range occupied by human voices.

There are regions of reading activation, notably in superior temporal cortex, that do not overlap with topological maps; and there are similar, non‐overlapping regions in frontal cortex near the pars triangularis region. Whether or not these regions are specialized for purely linguistic processing is an important question for future research. Some recent evidence suggests that these regions may have specific functions beyond language processing [Dick et al., 2011].

We found increased activation specific to reading meaningful paragraphs in occipital cortex and adjacent regions in inferior/middle temporal lobes and in the fusiform gyrus. Although these regions are not often considered as significant for language comprehension, empirical evidence from lesion studies [Rubens and Kertesz, 1983] and invasive electrophysiology [Burnstine et al., 1990; Krauss et al., 1996; Luders et al., 1991; Mani et al., 2008] suggests that the 'basal temporal language area' in the inferior temporal lobe and fusiform gyrus is as strongly linked with language functions as classical language areas. Electrical stimulation of these regions not only interferes with reading and language understanding but also often leads to speech arrest. Judging from published reports, this area may overlap some of our inferotemporal retinotopy. In addition, in the aphasiology literature, a prominent aphasia called the transcortical sensory aphasia, where the patient exhibits poor “Wernicke'‐like comprehension”, is associated with brain lesions most often in inferior temporal cortex, sometimes including the basal temporal language area, but commonly also involving visual areas in the lateral aspect of the adjoining occipital lobe [Kertesz et al., 1982; Sharp et al., 2004]. It is interesting in this context that picture “lexical items” inserted into text sentences can be effortlessly integrated into ongoing linguistic discourse comprehension, even at faster‐than‐normal reading speeds [Potter et al., 1986]. Our results show that the regions activated during text comprehension in occipital cortex encompass a number of distinct visual areas, both lower‐level (such as V1 and V2) as well as higher‐level (such as ventral V3, MT, V8, posterior inferotemporal cortex, and LIP+). Taken together, this psychological, brain lesion, and neuroimaging data suggests that some aspects of linguistic meaning assembly may be taking place within intermediate level visual areas. It should be noted that not all activation observed in the occipital cortex for English vs. Fixation was significant for English vs. Hindi contrast. It was instructive to find out where these activations (for English vs. Hindi) lie relative to well‐defined distinct retiniotopic maps in posterior occipital cortex.

In contrast to the substantial overlap with visual and auditory maps, somewhat surprisingly, we found rather less overlap of reading activations with somatomotor maps. We did not attempt to select semantic content in our reading task specific for any specific sensory modality and used simple informative passages dealing with natural phenomena, science, information about famous people and paintings, and so on; this may have resulted in less somatosensory content than visual and auditory content. A future direction would be to see if modality specific content modulated the amount of measured overlap with particular modality‐specific topological maps. For somatomotor maps, the primary overlap zone was a frontal multimodal overlap zone adjoining primary motor cortex (see next).

There were multisensory maps in several locations including lateral prefrontal and in the extreme superior and posterior part of the lateral sulcus near the supramarginal gyrus. The frontal multisensory overlap zone also overlapped reading activation, but the posterior lateral sulcus zone did not. Note that we have defined “multisensory” in the restricted sense of voxels that contain topological maps in more than one modality (not voxels that merely respond to more than one modality).

The pattern of activations for the reading experiment across the whole cortex is similar to what was found in a recent natural reading experiment reported by Choi et al. [2014]. Compared to Choi et al., our study utilized controlled/matched saccades as opposed to free eye movements, and a higher resolution, fully surface‐based analysis stream as opposed to a volume‐based cross‐subject analysis only mapped to an average surface at the end. As with our study, the activation patterns in Choi et al. exhibited eye movement related activation patterns in IPS, the FEF, and the dorsomedial FEF, as well as unique activation for text in more anterior IFG. As observed in our study, Choi et al. also report no activations during natural reading in somatosensory/motor cortex. Visual activation—likely within retinotopic areas judging from our data—was observed even when comparing text with pseudo‐words—a closer comparison than ones we used.

There is often an implicit assumption that whatever is going on in higher‐level visual areas can only count as being involved in language comprehension if it were equally activated by an auditory language understanding task—that is, by this definition, there can be no modality‐specific involvement in language comprehension. Given how poorly understood the cortical neural activity patterns underlying language comprehension currently are, we think it is important to keep an open mind about modality specificity or functional duplication. It seems quite possible that, for example, anaphor processing may employ modality‐specific representations; the cortical computations and cortical areas involved in dereferencing an auditory “that” could well be different than for resolving a printed “that” that appeared in a particular location on the page. It is difficult to directly experimentally address the question of modality‐specific language activations. For example, simultaneous, retinotopically overlapping visual tasks, widespread functional disruption of higher visual areas (e.g., by transcranial magnetic stimulation), or widespread lesions to higher visual areas would disrupt naturalistic reading comprehension itself—the object of study.

Overall, our results show that topological sensory‐motor maps, which play crucial functional roles in primary sensory and motor areas, are also found in some of the brain regions involved in a higher‐level cognitive function such as reading comprehension. This study provides the first comprehensive overview on the interface between a high‐level complex cognitive process reading comprehension, and topologically organized visual, auditory, and somatomotor representations. Determining the specific roles played by the regions identified here remains an important question for future research in the field of topological sensory‐motor maps as well as language.

## Supporting information

Supporting Information Figure 1.

Supporting Information Figure 2.

Supporting Information Audio 1.
