# Supplementary figures and images for: Areas activated during naturalistic reading comprehension overlap topological visual, auditory, and somatotomotor maps
Source: Hum Brain Mapp. 2016 Apr 7;37(8):2784–810. doi: 10.1002/hbm.23208 (PMC4949687; doi:10.1002/hbm.23208)

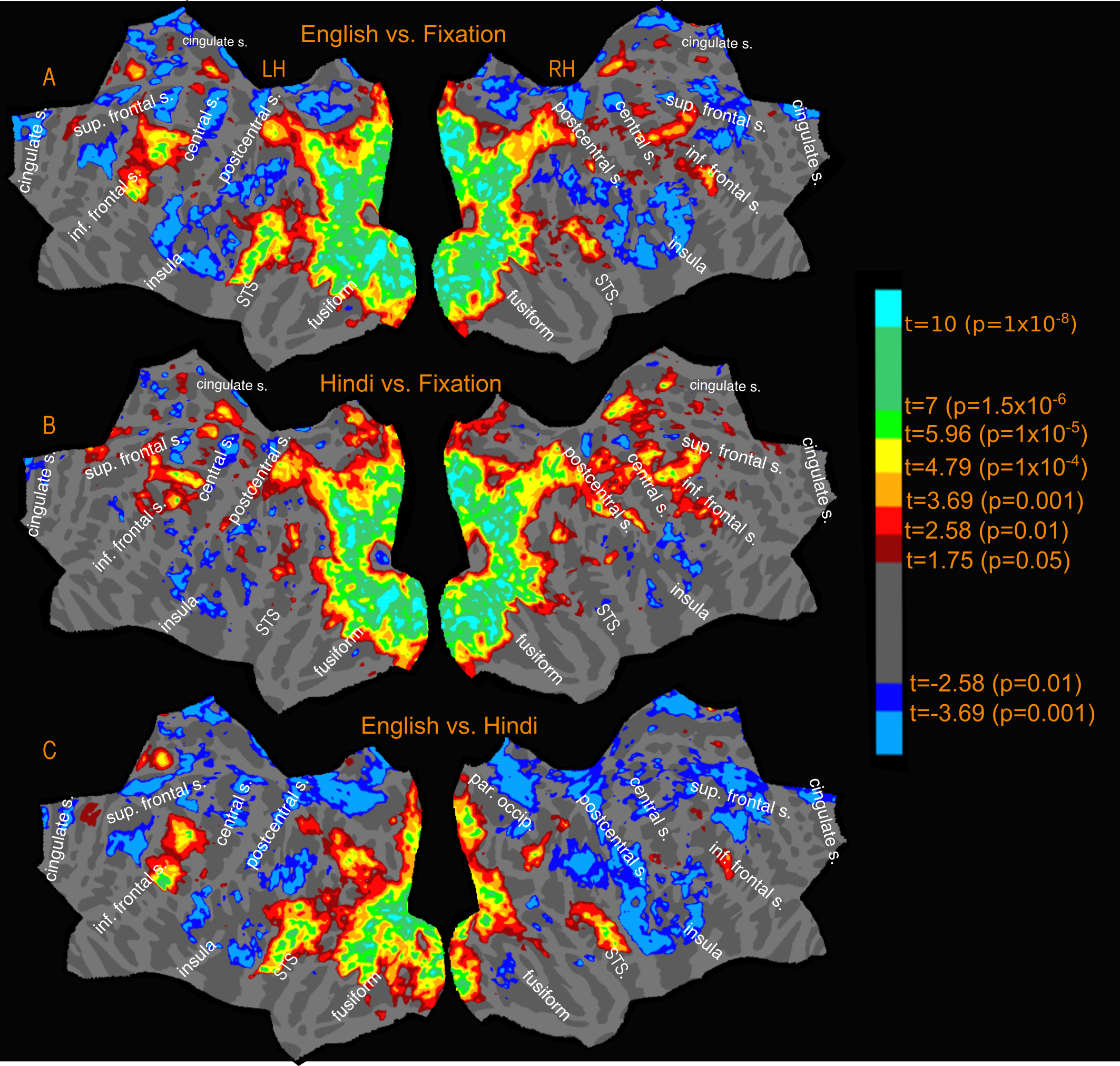

Supplement: Supplementary file 1 — Supporting Information Figure 1. [file HBM-37-2784-s001.tiff]

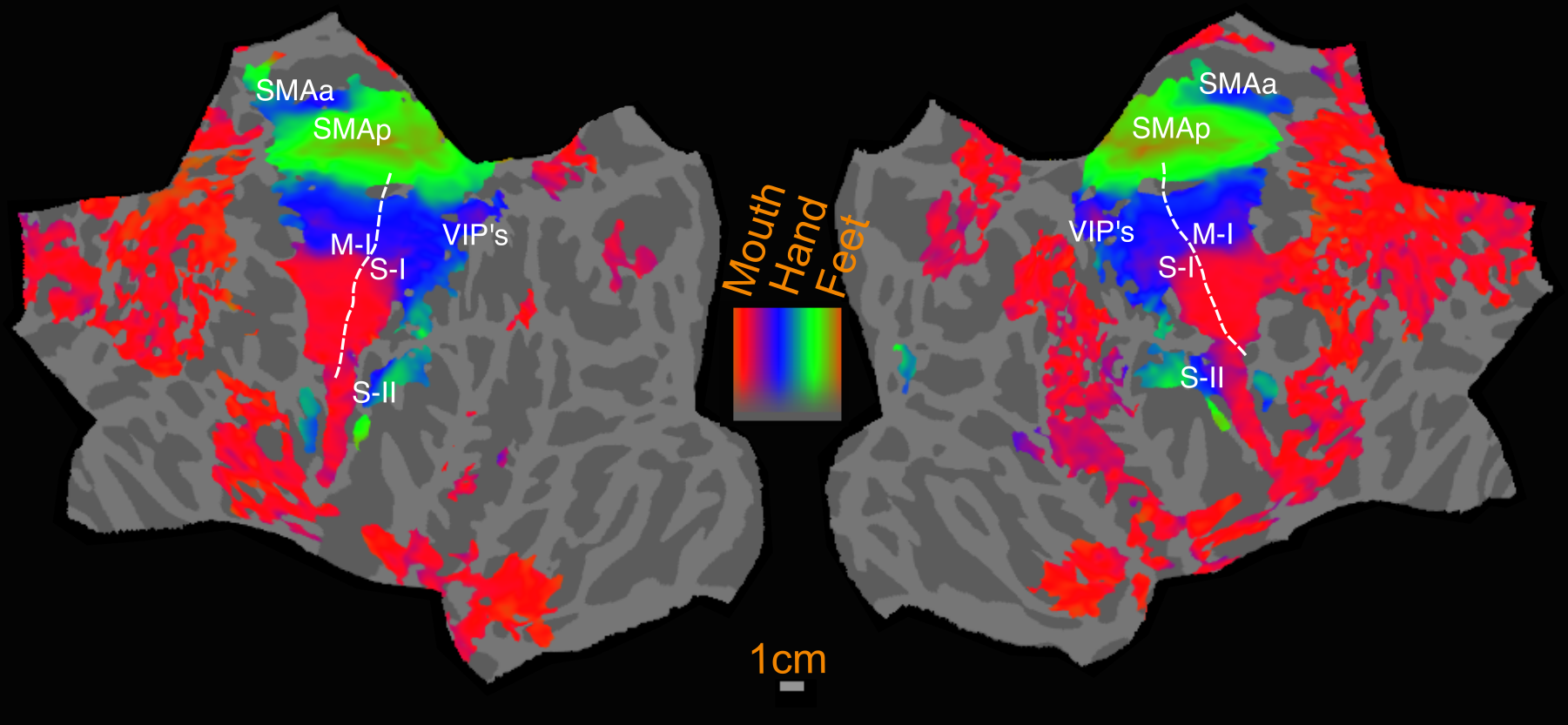

Supplement: Supplementary file 2 — Supporting Information Figure 2. [file HBM-37-2784-s002.tiff]
